# Evaluation of the Effects of Harmine on β-cell Function and Proliferation in Standardized Human Islets Using 3D High-Content Confocal Imaging and Automated Analysis

**DOI:** 10.3389/fendo.2022.854094

**Published:** 2022-07-04

**Authors:** Alexandra C. Title, Maria Karsai, Joan Mir-Coll, Özlem Yavas Grining, Chantal Rufer, Sebastian Sonntag, Felix Forschler, Sayro Jawurek, Thomas Klein, Burcak Yesildag

**Affiliations:** ^1^ Diabetes Research, InSphero AG, Schlieren, Switzerland; ^2^ Department of Cardio-Metabolic Diseases, Boehringer Ingelheim Pharma GmbH & Co. KG, Biberach an der Riß, Germany

**Keywords:** diabetes, islets, pancreatic β-cell, proliferation, 3D high-content imaging, islet microtissues, spheroids

## Abstract

Restoration of β-cell mass through the induction of proliferation represents an attractive therapeutic approach for the treatment of diabetes. However, intact and dispersed primary islets suffer from rapidly deteriorating viability and function *ex vivo*, posing a significant challenge for their experimental use in proliferation studies. Here, we describe a novel method for the assessment of compound effects on β-cell proliferation and count using reaggregated primary human islets, or islet microtissues (MTs), which display homogeneous size and tissue architecture as well as robust and stable functionality and viability for 4 weeks in culture. We utilized this platform to evaluate the dose-dependent short- and long-term effects of harmine on β-cell proliferation and function. Following compound treatment and EdU incorporation, islet MTs were stained and confocal-imaged for DAPI (nuclear marker), NKX6.1 (β-cell marker), and EdU (proliferation marker), allowing automated 3D-analysis of number of total cells, β-cells, and proliferating β- and non-β-cells per islet MT. In parallel, insulin secretion, intracellular insulin and ATP contents, and Caspase 3/7 activity were analyzed to obtain a comprehensive overview of islet MT function and viability. We observed that 4-day harmine treatment increased β- and non-β-cell proliferation, NKX6.1 expression, and basal and stimulated insulin secretion in a dose-dependent manner, while fold-stimulation of secretion peaked at intermediate harmine doses. Interestingly, 15-day harmine treatment led to a general reduction in harmine’s proliferative effects as well as altered dose-dependent trends. The described methodology provides a unique tool for *in vitro* high-throughput evaluation of short- and long-term changes in human β-cell proliferation, count and fraction along with a variety of functional parameters, in a representative 3D human islet model.

## Introduction

Both type 1 (T1D) and type 2 diabetes (T2D) pathophysiologies involve a loss of functional β-cell mass through a combination of β-cell apoptosis (1–3) and dedifferentiation (4–6). Autopsy studies have reported deficits in β-cell mass of up to ~99% in T1D patients, and up to ~65% in T2D patients. Importantly, such studies have suggested that even decades after diagnosis, T1D patients typically maintain a small number of residual β-cells (3, 5, 7–11). Restoration of β-cell mass, through the induction of proliferation of residual endogenous β-cells, therefore represents a promising treatment strategy for the recovery of glucose homeostasis in diabetic patients.

Human β-cell proliferation follows consistent patterns according to developmental stage and age in humans. Ki67-labeling analysis in postmortem pancreatic tissues has indicated that the highest proliferative rates occur during the perinatal period, with ~2-3.5% of β-cells proliferating, followed by a rapid decline in proliferation during the first two years of life, approaching a rate of <0.5% in adults that continues to decrease with age (12–15). Epigenomic and transcriptomic analyses have suggested this recalcitrance to replication in adult β-cells to be correlated with repressive histone marks and increased methylation in the promoter regions of cell-cycle related genes, as well as increased expression of senescence markers in adult vs. juvenile β-cells (10, 16–18).

Most studies investigating β-cell proliferation have been performed in rodent models, leading to greater knowledge of the signaling pathways controlling β-cell replication in mice than in humans (19). Human and rodent islets, however, not only exhibit intrinsic differences in structure, composition, and function, but also in proliferative capacity (20–22). While rodent β-cells follow similar development- and age-related trends as in humans, rodent β-cells have much higher proliferative rates (10-30% neonatally and 1% in adulthood) (22–24). Intriguingly, during periods of high metabolic demand in adulthood, including pregnancy and obesity, β-cell mass increases significantly in both rodents and humans. While rodent studies point to a mechanism of increased β-cell proliferation (25, 26), robust data demonstrating increased proliferation rather than neogenesis in humans during pregnancy or obesity is still lacking (27, 28), and the precise contribution of proliferation remains controversial (22, 29–31). As a further illustration, human islets transplanted into mice fed a high-fat diet do not proliferate whereas endogenous mouse islets do (22, 31). These examples highlight potential fundamental differences in β-cell proliferative capacity between rodents and humans and emphasize the need to focus on human islets for the study of β-cell proliferation. Interpretation of these discrepancies is further obscured by the fact that most studies in rodents are performed in juvenile animals, whereas human studies are typically performed using material from older adults. For these reasons and perhaps others, a majority of agents that promote β-cell proliferation in rodents have proven unsuccessful in human islets (10, 23). A promising exception is the class of dual-specificity tyrosine phosphorylation-regulated kinase 1A (DYRK1A) inhibitors, which have recently been identified as an effective strategy to induce β-cell-proliferation in both rodent and human islets (32–36). Harmine, an orally bioavailable, naturally occurring compound, is the best studied and a highly potent DYRK1A-inhibitor (36), and has been found to induce human β-cell-proliferation up to 3% (10, 37).

Due to the frequent lack of translatability of proliferative agents identified in rodent studies to human, it is imperative that new methods be developed to enable the study of β-cell proliferation directly in human islets. Recent advances have led to the development of EndoC-βH human β-cell lines that replicate human β-cell function and gene expression patterns remarkably well, as well as recapitulate low proliferation rates after excision of immortalization transgenes (38–40). While representing important tools for research, the EndoC-βH cell lines are derived from fetal pancreas and may thus not model adult human β-cells and proliferative capacity to a sufficiently translatable level. Isolated human islets remain the gold standard for the study of human β-cells; their experimental use, however, is remarkably challenging due to the striking variation in islet size, cellular composition, function, and purity (41), as well as low viability and functionality in prolonged culture, especially of large islets (42). These challenges can be addressed through enzymatic dispersal and 2D monolayer culture, although the lack of organotypic cell-to-cell and cell-to-extracellular matrix interactions can have profound effects on proliferation: adult dispersed islets dedifferentiate and hyperproliferate in culture (43), and the extracellular matrix used for culture can also strongly influence β-cell proliferative capacity (44). In contrast, human pseudoislets, or islet microtissues (MTs), generated by gentle enzymatic dissociation and scaffold-free reaggregation of human native islets, represent a uniform, functionally robust and long-lived *in vitro* islet model. Islet MTs preserve a native-like endocrine cell distribution and composition, remain glucose-responsive for at least 28 days, and due to their high standardization, are amenable to high-throughput screening (45, 46).

In the current study, we describe the development of an automated three-dimensional (3D) staining, high-content imaging, and image analysis platform utilizing human islet MTs for the assessment of changes in proliferation rates and total numbers of β- and non-β-cells. We used the established platform to evaluate harmine’s dose-dependent effects across multiple donors and for different treatment durations. In parallel we analyzed insulin secretion, intracellular insulin and ATP contents, and Caspase 3/7 activity to understand harmine’s effect on islet MT function and viability. The strength of the described platform lies in our ability to evaluate short- and long-term compound effects based on diverse microscopic and functional endpoints, as well as the unique possibility for the assessment of β- and non-β-cell count over time, all within a highly standardized and relevant 3D primary human islet model.

## Materials and Methods

### Human Islet Microtissues

Human islets were purchased from Prodo Laboratories Inc. (Irvine, CA). All islets were obtained from deceased donors following consent from next of kin. Donor information can be found in **Table 1**. To generate 3D InSight™ Islet MTs (InSphero, AG), human islets were dispersed in dissociation solution (1X TrypLE™ Express solution - Thermo Fisher Scientific #12604013, with 40 µg/ml DNase I - Sigma-Aldrich #10104159001) by gentle pipetting for 10 min at 37°C. Remaining cell clumps were filtered through a cell strainer of 70 μm pore size. Approximately 1700 dispersed live islet cells were reaggregated in each well of the Akura™ PLUS Spheroid Hanging Drop System for 5 days (InSphero AG, CS-06-001-02), to achieve a volume of roughly 1 islet equivalent (IEQ) per MT, which corresponds to an islet with 150 µm diameter. The reaggregated islets were transferred to and cultured in Akura™ 96 Spheroid Microplates (InSphero AG, CS-09-001-03) with 3D InSight™ Human Islet Maintenance Medium (InSphero AG, CS-07-005-01). Islet MT cultures were maintained at 37°C in a humidified atmosphere containing 5% CO_2_, and cell culture medium was exchanged every 2-3 days.

**Table 1:** Information from Donors used for MT production.

Donor number in paper	UNOS ID #	Donor Sex	Donor age (years)	Donor BMI (kg/m^2^)	Donor HbA1c	Islet Isolation Center	Donor history of diabetes?
1	AIGQ368	Male	25	26.5	5.8%	Prodo Laboratories	No
2	AIIY018	Male	39	29	5.4%	Prodo Laboratories	No
3	AIIE165	Male	29	23.1	5.3%	Prodo Laboratories	No

For each biospecimen collection, a donor consent form is available that documents that the next-of-kin consented to collection, transfer and use of human biospecimens and data for research purposes, including the possible transfer of the human biospecimens to pharmaceutical and biotech companies.

### Harmine and 5-Ethynyl-2′-Deoxyuridine Treatment

For the 4-day experiments, islet MTs were treated with harmine and EdU for 4 days, and for the 15-day experiments, islet MTs were treated with harmine for 15 days and EdU for the final 4 days. For the washout experiments, islet MTs were treated as described for the 15-day experiment, followed by two washes with fresh media and further incubation without harmine for 4 days. During the course of the treatment, fresh medium was supplied every 2 to 3 days, and plates were dosed using a Tecan D300e Digital Dispenser (Tecan) with 0**–**10 µM harmine (Sigma, 286044) with or without 10 µM EdU (ThermoFisher, C10357), with DMSO normalization across all wells (to the highest volume).

### Immunofluorescence Staining

Islet MTs were fixed with 4% PFA, permeabilized with 0.5% Triton X-100 (in PBS) and incubated in Click-It EdU reaction cocktail (ThermoFisher, C10357) according to manufacturer’s instructions. Following blocking in 10% FCS, MTs were incubated with rabbit anti-NKX6.1 (Abcam, ab221549) overnight and goat anti-Rabbit Alexa Fluor 568 (ThermoFisher, A-11036) and DAPI (Sigma, D9542) (1 μg/mL) for 4 hours in antibody incubation buffer (10% FCS, 0.2% Triton X-100 in PBS). Nonspecific binding was removed with repeated wash steps in 0.2% Triton X-100 in PBS after both antibody incubations. Stained MTs were then transferred to Akura™ 384 Spheroid Microplates (InSphero AG, CS-09-003-02) and cleared with ScaleS4 (40 w/v% D-(-) sorbitol, 10 w/v% glycerol, 4M Urea, 0.2% w/v% Triton X-100, and 15 v/v% DMSO in MilliQ water) (47).

### Confocal Imaging

Cleared MTs were imaged in black-walled, thin-bottomed Akura™ 384 Spheroid Microplates (InSphero AG, CS-09-003-02). Imaging was performed on a Yokogawa CQ1 confocal benchtop high-content analysis (HCA) system (Yokogawa Electric Corp., Tokyo, Japan) using a 40x dry objective (Olympus, Tokyo, Japan). Image stacks were acquired at a Z-step size of 3 µm to encompass the whole MT. This step size was selected to maximize the segmentation quality of the nuclear markers and the reliability of colocalization analysis.

### Image Analysis

The analysis of the confocal image stacks was performed using the Yokogawa CellPathfinder software (version 3.04.03.02). Each image was segmented and analyzed for nuclear count (DAPI), β-cell count (NKX6.1), proliferating cell count (EdU), and proliferating β-cell count (EdU colocalized with NKX6.1). The analysis algorithm consisted of 4 steps. In the first step, the whole spheroid region was determined based on the DAPI channel using Otsu thresholding. In the second, third and fourth steps, nuclei, β-cells and proliferating cells were segmented and labeled individually in the DAPI, NKX6.1 and EdU channels, respectively, using a dynamic thresholding method. In the final step, proliferating β-cells were identified based on the colocalization of labels marking β-cells and proliferating cells. Accuracy of counts was verified with manual counting in a subset of islet MTs.

### Analysis of ATP Content, Caspase-3/7 Activity, and Secreted and Intracellular Insulin

Culture medium was collected from wells to determine chronic insulin secretion over the course of 24–72 hours, depending on the experiment. MTs were then washed twice with Krebs Ringer Hepes Buffer (KRHB – 131 mM NaCl, 4.8 mM KCl, 1.3 mM CaCl_2_, 25 mM Hepes, 1.2 mM KH_2_PO_4_, 1.2 mM MgSO_4_, 0.5% BSA) containing 2.8 mM glucose and equilibrated for 1 hour at 37°C in this buffer. Basal and stimulated insulin secretion were measured from the supernatants collected after 2-hour incubation of MTs in KRHB containing 2.8 mM glucose or 16.7 mM glucose, respectively. MTs were then lysed using the CellTiter-Glo^®^ Luminescent Cell Viability Assay (Promega, G9241) supplemented with protease inhibitor cocktail (Promega, G6521) for total ATP and insulin content measurement. Insulin concentrations were analyzed from the harvested supernatants and lysates by using the Stellux Chemi Human Insulin ELISA (Alpco, 80-INSHU-CH10) or the Insulin Ultra-Sensitive Assay kit (Cisbio, 62IN2PEG). Caspase 3/7 activity was measured from a separate set of harmine-treated MTs using the Caspase-Glo^®^ 3/7 Assay (Promega, G8090) according to manufacturer’s instructions. As a positive control for this assay, we treated MTs for 4 days in parallel with cytokines: IL-1β (Sigma-Aldrich #I17001, 20 ng/mL), IFNγ (Sigma-Aldrich #I3265, 100 ng/mL), and TNFα (Sigma-Aldrich #PHC3016, 100 ng/mL). Luminescence was measured using a Tecan Spark 10M Microplate Reader (Tecan).

### Statistical Methods

Data are presented as mean ± standard error of the mean (SEM). Outlier detection was performed using Robust regression and outlier removal test (ROUT) with false discovery rate less than 5%. No outlier test was performed for proliferating cell fractions. Statistical significance was determined by one-way ANOVA followed by Dunnett’s *post hoc* analysis or by Student’s t-test, rejecting the null hypothesis at p < 0.05. Statistical significance is represented as * p < 0.05, ** p < 0.01, and *** p < 0.001.

## Results

### Establishing a High-Throughput-Compatible Platform for the Assessment of Human Islet Cell Proliferation and β-Cell Function in 3D

**Figure 1:**
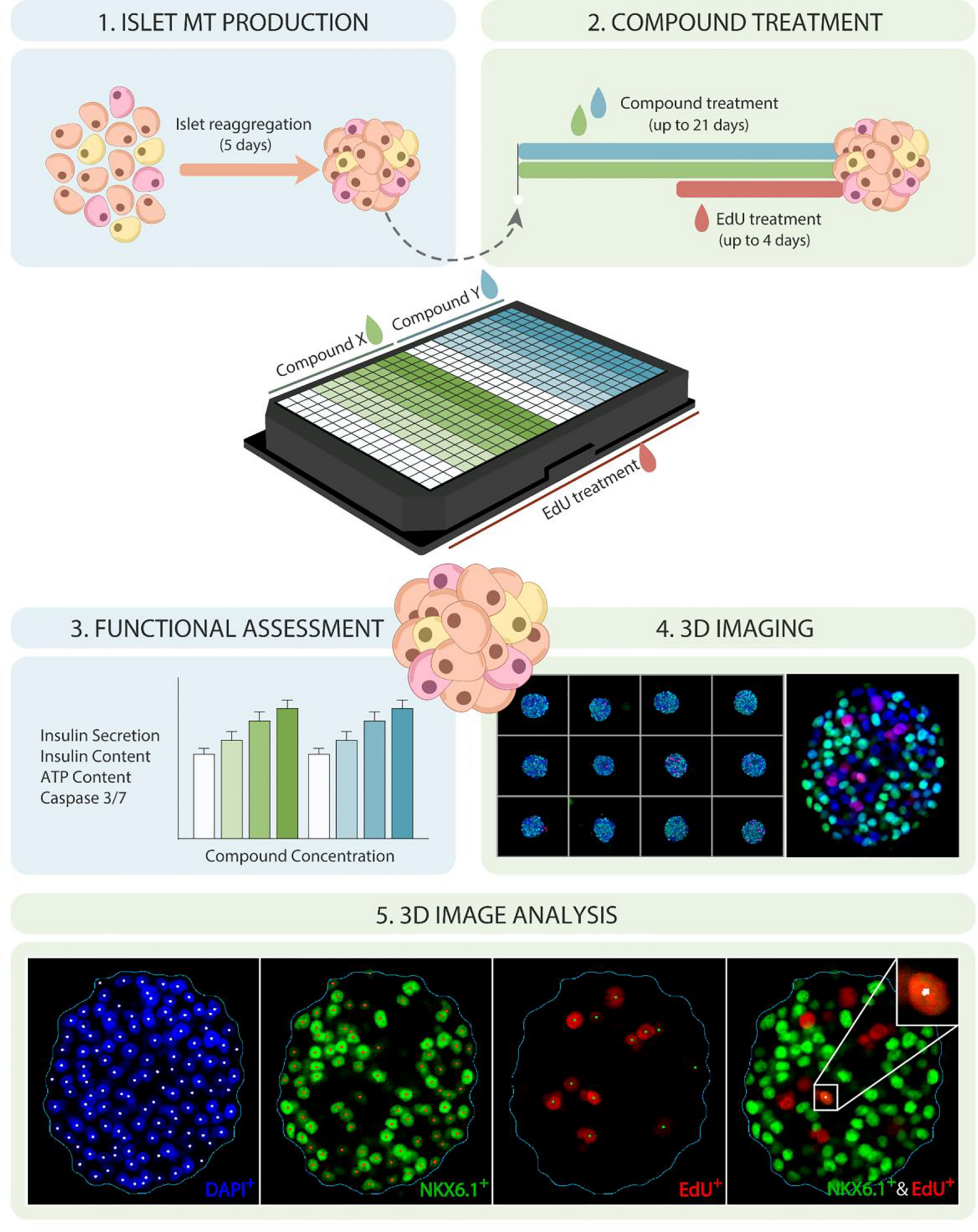
Establishing a high-throughput platform for evaluating β-cell proliferation and function in human islet MTs. Schematic representation of experimental timeline and platform for 3D evaluation of β-cell proliferation and function in human islet MTs. Dispersed islets are reaggregated into islet MTs for 5 days, followed by proliferative compound treatment for up to 21 days, including 5-ethynyl-2′-deoxyuridine (EdU) incorporation during the last 4 days of treatment. Islet MTs are then used for functional endpoints such as glucose-stimulated insulin secretion (GSIS), total insulin content, ATP content, and Caspase 3/7 activity, or fixed with 4% PFA. Fixed MTs are stained for DAPI, EdU, and NKX6.1, and imaged with a confocal, high-content imaging system. Image quantification is then performed to quantify DAPI-, NKX6.1-, EdU and EdU/NKX6.1-positive nuclei.

The goal of this study was to establish a high-throughput platform for the identification and validation of β-cell proliferative agents using a biologically relevant 3D human islet model, based on 3D high-content imaging and analysis, paired with functional endpoints. The experimental pipeline (**Figure 1**) begins with the generation of islet MTs from primary human islet cells by enzymatic dissociation and reaggregation in hanging drops, leading to the generation of human islet MTs with standardized size, composition, and function (45). After 5 days of aggregation, islet MTs are released into low attachment microwell plates and cultured for a minimum of 2 days before compound treatment. Proliferating cells are labelled with EdU (5-ethynyl-2′-deoxyuridine), a thymidine analog that is incorporated into replicating DNA. Since EdU can have adverse effects such as slowdown of cell cycle progression, its incorporation is limited to the final 4 days of long-term compound treatment to minimize EdU toxicity (48). Following compound and EdU treatment, islet MTs are either used for the assessment of islet function and viability through the analysis of glucose-stimulated insulin secretion (GSIS), intracellular insulin and ATP content, and Caspase 3/7 activity, or for the assessment of cellular proliferation through 3D high-content microscopy. For microscopic analysis, islet MTs are stained and confocal-imaged for DAPI, EdU and NKX6.1 in 3D. NKX6.1, a key transcription factor for the development and maintenance of β-cell identity, was selected as β-cell marker due to its high β-cell-specificity (49) and its nuclear localization, which facilitates downstream co-localization analysis with EdU and DAPI. Stained MTs are then cleared and imaged in Z-stacks encompassing the whole MT. Finally, image analysis is carried out to determine total cell number, β-cell count and β-cell fraction, and percentage of proliferating β- and non-β-cells through the quantification of DAPI, NKX6.1, and EdU signals. The first step of our analysis pipeline is spheroid detection based on the DAPI channel, enabling analysis throughout and limited to the MT. Within the detected spheroid, DAPI-, NKX6.1- and EdU-positive nuclei can be counted in a precise and automated fashion. For the detection of proliferating β-cells, nuclei with co-localized EdU and NKX6.1 signal are identified.

### Harmine Treatment Exerts Dose- and Donor-Dependent Effects on Proliferation and β-Cell Fraction in Human Islet MTs

**Figure 2:**
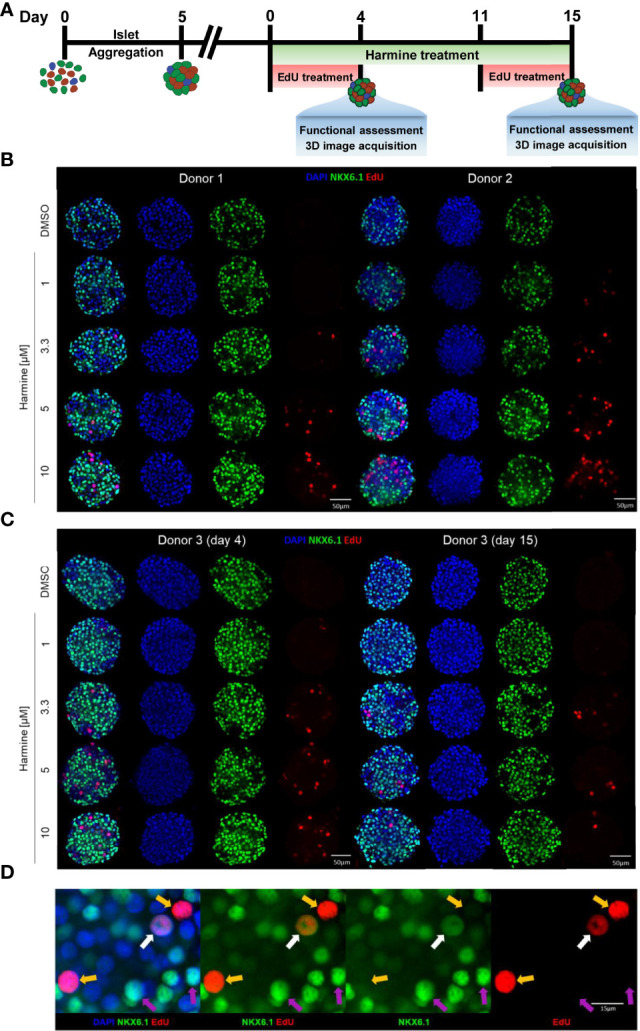
EdU and NKX6.1 labeling reveals dose and donor dependence of harmine-induced proliferation in human islet MTs. **(A)** Schematic representation of islet MT harmine treatment scheme. **(B, C)** Representative immunofluorescence images of islet MTs generated from three different donors, treated with DMSO or harmine at a concentration of 1, 3.3, 5, or 10 μM, for a duration of **(B)** 4 days for Donors 1 and 2 or **(C)** 4 days or 15 days for Donor 3. Scale bar, 50 μm. **(D)** Representative immunofluorescence image of a proliferating β-cell (white arrow) and proliferating non-β-cells (yellow arrow). Purple arrows indicate non-proliferating β-cells for comparison. **(D)** Scale bar, 15 μm. Images were generated from central Z-stacks and with the same thresholding settings within each donor.

**Figure 3:**
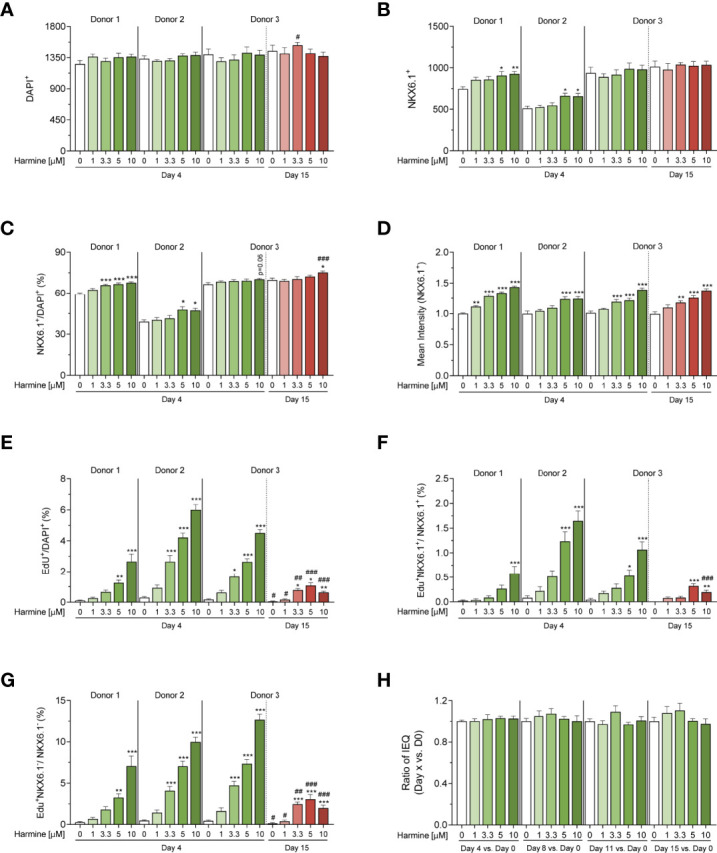
3D microscopy image analysis of harmine-induced proliferation in human islet MTs generated from 3 donors. **(A–F)** Quantification of **(A)** total cell number (DAPI-positive cell count), **(B)** total β-cell number (NKX6.1-positive cell count), **(C)** percent β-cell fraction (% NKX6.1/DAPI), **(D)** mean NKX6.1 intensity, **(E)** percent total proliferating cells (% EdU/DAPI), **(F)** percent proliferating β-cells (% EdU^+^NKX6.1^+^/NKX6.1^+^), **(G)** percent proliferating non-β-cells (% EdU^+^NKX6.1^-^/NKX6.1^-^), and **(H)** volume differences over time (Donor 3, 15-day treatment). **(A–H)** Data are represented as mean ± SEM, n = 6–12. Significance was determined by one-way ANOVA followed by Dunnett’s multiple comparisons test vs. the respective DMSO control (*p < 0.05, **p < 0.01, ***p < 0.001), or by student’s t-test for D15 vs. D4 comparisons between equal harmine doses (^#^p < 0.05, ^##^p < 0.01, ^###^p < 0.001).

As a validation of our newly established β-cell proliferation platform, we sought to determine the dose-dependency of harmine-induced proliferation in human islet MTs generated using islets from three different donors, with EdU incorporation as a measure of cumulative proliferation over 4 days. Our goal was thus to (1) provide proof-of-concept data utilizing our 3D β-cell proliferation platform (2), gain further insights regarding donor-to-donor variability in harmine-induced proliferative response and (3) correlate proliferation data with functional analysis. Islet MTs were treated with harmine at concentrations of 0, 1, 3.3, 5, or 10 μM for a duration of 4 days for short-term or 15 days for long-term experiments, with 10 μM EdU included during the final 4 days of treatment to label proliferating cells (**Figure 2A**). MTs were stained with NKX6.1 and labeled with DAPI and EdU, and Z-stacks were imaged throughout the whole MT (**Figures 2B–D**). Representative videos of 3D images encompassing the islet MTs are available in the **Supplementary Information** and provide valuable spatial information on NKX6.1 and EdU signal distribution with and without harmine treatment (**Supplementary Video S1**). Confocal images were then analyzed in 3D to perform comprehensive quantification of β-cell and proliferating cell populations (**Figures 3A–H** and **Supplementary Videos S2**, **S3**). On average, 1405 DAPI-positive cells were quantified per MT, thus representing a majority of the cells seeded in each islet MT (**Figure 3A**). Analysis of NKX6.1-positive cells revealed a β-cell fraction of 40-65% (**Figures 2B, C**, **3C**).

In the short-term (4-day) treatment group, DMSO-treated MTs exhibited little to no proliferation (on average, 2.7 proliferating cells per MT, constituting 0.2% total proliferation), whereas harmine stimulated proliferation in all donors in a dose-dependent manner, achieving total islet proliferation rates of up to ~6% (% EdU^+^/DAPI^+^) (**Figures 2B, C**, **3E**). Reflecting this dose-dependent increase in proliferation, total DAPI count tended to increase slightly with harmine treatment compared to solvent (**Figure 3A**). Proliferation of both β-cells (**Figure 3F**) and non-β-cells (**Figure 3G**) was induced in a dose-dependent fashion. The proliferative rate of non-β-cells under harmine treatment was 5- to 10-fold higher than that of β-cells in the 3 donors studied, depending on the harmine dose. Intriguingly, β-cell number and β-cell fraction increased mildly with elevated harmine concentrations, with an observed increase in β-cell count (182 more β-cells for Donor 1 and 61 more β-cells for Donor 2 after 10 µM harmine treatment) that was significantly higher than the number of proliferating β-cells (5 β-cells for Donor 1 and 10 β-cells for Donor 2 after 10 µM harmine treatment) (**Figures 3B, C**). Finally, we observed a consistent, dose-dependent increase in mean NKX6.1 staining intensity with harmine treatment across all donors, (**Figures 2B–D**, **3D**). Of note, this increased intensity was likely restricted to non-proliferating β-cells as we observed that NKX6.1 expression levels were generally reduced in EdU/NKX6.1 double-positive cells (**Figure 2D**, white arrow vs. purple arrows).

Human islet MTs retain function and viability for a minimum of 21 days after aggregation, thus permitting longer treatment schemes than those feasible with native islets (45). To explore the effects of harmine during longer treatment periods, we also exposed Donor 3 MTs to harmine for 15 days. We observed a significant drop in both β- and non-β-cell proliferation rates following 15-day continuous harmine treatment of islet MTs from Donor 3 compared to the 4-day treatment. Furthermore, an interesting change in proliferative pattern was observed: while 10 μM harmine induced the maximal rate of proliferation after 4 days of treatment, the proliferative peak was achieved with 5 μM harmine after 15 days of treatment (**Figures 2C**, **3E–G**). Total DAPI count was similar in the solvent-treated MTs from Donor 3 after 4 and 15 days of treatment, illustrating the stability of islet MT size over time (**Figure 3A**). In conjunction with the higher proliferation rates at mid-concentrations of harmine, we observed a peak in DAPI count at 3.3 μM harmine after 15 days of treatment; in order to explore this further, we calculated relative volume changes at 3–4 day intervals and confirmed a correlation between moderate harmine dose and increased MT volume (**Figures 3A, H**). Interestingly, a greater increase in β-cell fraction was observed with 10 μM harmine after 15 days of treatment than after 4 days of treatment for Donor 3 **(** **Figure 3C**). Of note, EdU was only included during the final 4 days of harmine treatment (**Figure 2A**), and it is thus likely that the reduction in proliferation observed with 10 μM harmine treatment after 15 days of treatment (**Figures  2C**, **3E**) is not reflective of proliferative rates throughout the whole treatment period. Finally, long-term treatment with harmine led to increased NKX6.1 intensity in a dose-dependent manner that correlated well with 4-day treatment (**Figures 2C**, **3D**).

In order to confirm that the observed reduction in proliferation after 15 days of treatment was not due to the longer culture time of the MTs, we performed an additional, independent experiment with Donor 3 MTs, whereby we compared harmine treatments performed for the first 4 days vs. the last 4 days of the 15-day treatment period. This comparison revealed that proliferation rates remained unchanged between these two 4-day treatments, confirming that increased MT culture time was not the cause for the reduced proliferation observed after 15 days of harmine treatment (**Figure S1**).

### Harmine Treatment Exerts Donor-Dependent Effects on β-Cell Function but Not on Human Islet MT Viability

The high reproducibility in size and composition of human islet MTs enables complementing imaging analysis with functional studies using islet MTs from the same production lot. In order to determine the impact of harmine treatment on islet function and viability, as well as evaluate donor-to-donor variability, we subjected islet MTs generated from the same donors as those utilized for the high-content imaging analysis above to additional evaluation of insulin secretion, intracellular insulin and ATP content, and apoptotic activity following 4- or 15-day harmine treatment.

**Figure 4:**
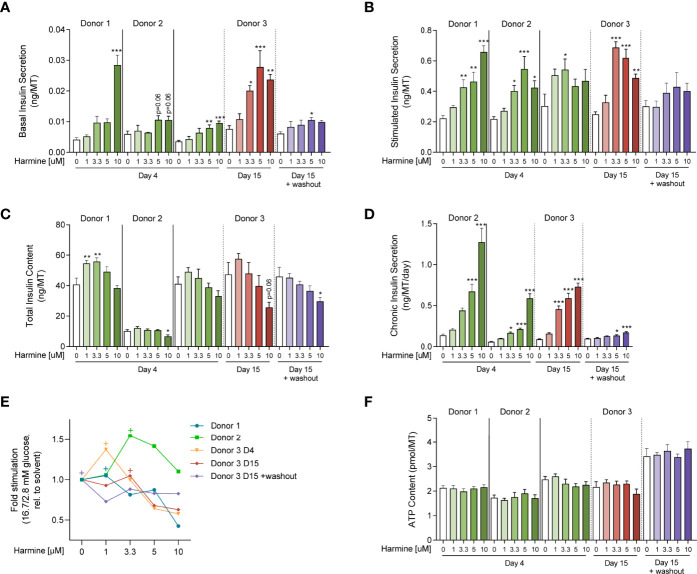
Evaluation of harmine dose-response on human islet MT function and viability. **(A)** Basal and **(B)** stimulated insulin secretion of islet MTs treated with **(A)** 2.8 mM glucose or **(B)** 16.7 mM glucose for 2 hours (ng/MT). **(C)** Total insulin content of islet MTs after GSIS (ng/MT). **(D)** Chronic insulin secretion of islet MTs over the course of 24–72 hours (ng/MT/day). **(E)** Stimulated-to-basal fold-stimulation (16.7/2.8mM glucose). “+”‘s indicate maximum fold-stimulation for each dataset. **(F)** ATP content of islet MTs (pmol/MT). **(A–D, F)** Data are represented as mean ± SEM, n = 5–12. Significance was determined by one-way ANOVA followed by Dunnett’s multiple comparisons test vs. the respective DMSO control (* p < 0.05, ** p < 0.01, *** p < 0.001).

Functionally, short-term harmine treatment led to an increase in basal insulin secretion in a loosely dose-dependent manner, with the highest dose of 10 μM inducing near-significant or significant increases in all 3 donors (**Figure 4A**). In parallel, glucose-stimulated insulin secretion was also increased with harmine treatment, although the harmine concentration inducing the highest insulin stimulation varied between 3.3, 5, and 10 μM depending on the donor (**Figure 4B**). Interestingly, total insulin content per MT tended to be higher at low doses of harmine (1, 3.3 μM) compared to the solvent control, while this trend was reversed at higher concentrations of harmine (10 μM) (**Figure 4C**). In order to determine the source of insulin depletion with higher harmine concentrations, we evaluated insulin secretion and accumulation in the culture medium in 2 donors, which revealed a dose-dependent increase in chronic insulin secretion as evaluated during the final 24–72 hours of culture (**Figure 4D**). The reduced insulin content can at least be partially explained by the amount of insulin that accumulated in the culture medium. Stimulated-to-basal fold stimulation (16.7/2.8 mM glucose), a relevant marker of optimal β-cell function, peaked at 1 or 3.3 μM harmine concentrations depending on the donor (**Figure 4E**), and decreased after treatment with 5 and 10 μM harmine, suggesting that higher harmine concentrations may in fact reduce β-cell functionality compared to lower doses.

Long-term (15-day) treatment with harmine in Donor 3 led to similar functional effects as observed after 4 days of treatment: increased basal and stimulated insulin secretion, increased chronic secretion, and decreased total insulin content (**Figures 4A–D**), although these trends were more pronounced compared to short-term treatment. Interestingly, stimulated secretion followed a downward trend from 3.3 μM to 10 μM (**Figure 4B**). Peak fold-stimulation was achieved with 3.3 μM harmine after 15 days of proliferation (**Figure 4E**).

In order to determine whether functional changes induced by harmine are maintained in the absence of the compound, we performed 15-day treatment of MTs with harmine, followed by a 4-day washout period. Surprisingly, the observed increases in basal and stimulated insulin secretion were greatly reduced following the washout (**Figures 4A–B**), while chronic insulin secretion was almost completely normalized (**Figure 4D**). The trend of declining total insulin content with higher harmine doses was maintained, although to a reduced extent compared to 15-day treatment without the washout period (**Figure 4C**).

Finally, to determine the effects of harmine on viability and apoptosis, we analyzed ATP content and Caspase 3/7 activity. Neither ATP content nor Caspase 3/7 activity was significantly altered with harmine treatment after 4 or 15 days (**Figures 4F**, **S2A–C**), suggesting that harmine has little effect on islet viability and apoptosis.

## Discussion

Several β-cell proliferative compounds have been identified based on studies in animal models and cell lines, yet the effects of very few have been successfully translated to the primary human β-cell (50). Whether the adult human β-cell can proliferate *in vitro* is still under debate, as some studies failed to detect any proliferation whereas others observed rates at around 0.1% in a donor-dependent fashion (35, 44). It has also been reported that the proliferative capacity of the β-cell can be altered according to the extracellular matrix used for cell culture, which may explain the discrepancies between various studies (44). Therefore, in this study we strove to answer to the strong need for a standardized, physiologically relevant and long-lived 3D islet model for the assessment of compound effects on human β-cell proliferation and absolute count.

One of our principal goals was to establish an automated and high-throughput-compatible analysis pipeline of β- and non-β-cell proliferation and fraction in 3D. To do so, we have dedicated substantial resources in the optimization of 3D staining and tissue clearing methodologies suitable for our islet MTs. Establishment of 3D spheroid detection and automated DAPI and EdU quantification was straightforward with built-in algorithms in the CellPathfinder software. Quantification of NKX6.1-positive nuclei, however, posed an additional challenge due to the broad range in NKX6.1 expression in β-cells, and the observed downregulation in NKX6.1 intensity in proliferating β-cells, consistent with previous reports that describe lower expression of β-cell identity factors during proliferation (43, 51). Costaining with insulin would thus be beneficial in confirming the identity of these cells. NKX6.1 quantification was further complicated by the dose-dependent increase in intensity of NKX6.1 signal with harmine treatment. In order to avoid discrepancies in analysis between treatment groups, we truncated NKX6.1 signal to bring all wells within a similar range of intensities before applying dynamic thresholding for β-cell quantification. This thresholding approach was instrumental in obtaining precise β-cell quantification among different treatment groups and helped to reduce false positives and negatives. We have confirmed the precision of the automated detection of proliferating β-cells by manual count of proliferating β-cells for many of the tested conditions. The detected β-cell fraction (40-65%) fell within the expected range in humans (52).

As a proof-of-concept study, we conducted harmine dose response treatments to evaluate the performance of our platform and gain more insights regarding human donor-to-donor variability in proliferation and function in response to harmine. Several previous studies have investigated the proliferative potential of harmine in rodent and human islets. 10 μM harmine treatment was found to induce β-cell proliferation rates of ~0.25-2.5% (32, 33, 36, 37, 53, 54) in dispersed human islets *in vitro*, and rates of 0.8-2% were observed in human islets transplanted into immunodeficient mice treated with 10 mg/kg harmine (32, 36, 37), with some of the variation accounted for by differences in labeling method of proliferating cells (KI67, EdU) and β-cells (PDX1, NKX6.1, Insulin, C-peptide). Harmine also efficiently induced β-cell proliferation in our platform, with quite low donor-to-donor variation. The observed proliferative rates identified based on the quantification of EdU and NKX6.1 were within a similar range to those previously reported (32, 33, 36, 37, 53, 54). Our data also confirmed the non-specificity of harmine: we and others have found that harmine induces high levels of proliferation of non-β-cells (32, 36, 37, 53), although this has been suggested to be rectified through combinatorial treatment with GLP-1 receptor agonists (32). Perplexingly, one of the most interesting observations we made was the significantly increased β-cell count and fraction with high harmine dose treatment, despite low β-cell and high non-β-cell proliferation rates. The observed increase in β-cell count can only be partially explained by increased β-cell proliferation, pointing towards other mechanistic underpinnings for this observed increase in Donors 1 and 2.

Reaggregated human islet MTs can be maintained in culture for up to 4 weeks without loss of functionality, extending the treatment window from a few days to a few weeks compared to native human islets (45). Such a long treatment window could provide the opportunity to study the long-term effects of proliferative compounds, and even determine changes in MT volume and β-cell mass over time. In this study, we also sought to determine the effects of treating islet MTs with harmine over the course of 2 weeks. Surprisingly, proliferative rates were lower after 15 days of treatment compared to 4 days, and dose-related trends were altered. Of note, both 4-day and 15-day harmine treatment schemes included the same 4-day EdU incorporation period, thus eliminating the possibility of these effects being due to EdU-derived toxicity. These results suggest that long-term treatment with the selected harmine doses could in fact be detrimental towards proliferation. Interestingly, previously published dose-response curves generated using five different DYRK1A-inhibitors (harmine, INDY, Leu, 5-IT, and GNF) formed a bell-shaped curve of β-cell proliferation (55), indicating that excessive concentrations of such compounds do indeed inhibit proliferation. It would thus be interesting to determine whether a treatment scheme with intermittent washout periods would be beneficial for sustaining the potency of harmineor other DYRK1A-inhibitors. Finally, recent advances in single-cell sequencing have revealed that β-cells are heterogeneous and that certain subpopulations have a higher propensity to proliferate (56–58). It is thus also conceivable that proliferative rates were reduced after 15 days of treatment because the subpopulation of more readily proliferating β-cells had already replicated in the initial days of treatment.

While evaluation of the proliferative capacity of compounds such as harmine is crucial for determining their therapeutic potential, the parallel evaluation of islet function is equally important to ensure that the expanded β-cell population remains functional after compound treatment. We thus also investigated the dose-dependent effects of harmine on β-cell function and viability. Interestingly, previous studies only revealed maintenance of glucose-stimulated secretion in healthy human islets treated with 10 μM harmine for 72–96 hours (32, 36, 37), with little indication of increased secretion, although interpretation of results was often limited by the high variability associated with human islet experiments. Promisingly, harmine was found to amplify the insulin secretory response in islets isolated from T2D donors (32). Furthermore, human islets transplanted into diabetic mouse models treated with 10 mg/kg harmine led to improved glucose tolerance (36), suggesting functional effects of harmine *in vivo*. While the observed effects on β-cells likely contributed to this improvement, it is conceivable that these effects were not strictly islet-intrinsic, as harmine has also been found to induce adipose tissue browning and promote insulin sensitivity in adipose tissue (59, 60). In our isolated islet system, we were able to assess direct effects of harmine on islet function. Harmine induced increased basal, stimulated, and chronic insulin secretion across all donors tested, whereas total insulin content was reduced with higher harmine concentrations. Our data suggest this reduction in insulin content to be partially due to increased insulin secretion in non-stimulatory glucose concentrations. The effect of harmine on stimulated insulin secretion (up to a 3-fold increase) is intriguing, as it cannot be attributed to the proliferating β-cell population, which was too low in numbers (5-10 cells per MT) to account for the observed functional effects. This indicates that other mechanisms are likely involved. The increased overall NKX6.1 staining intensity observed in our system, likely accompanied by increased expression of other β-cell identity factors as observed by others (10, 32), may account for at least some of this improvement in β-cell function. However, at high concentrations, harmine also increased basal insulin secretion, an indicator of reduced β-cell maturity that has previously been associated with a proliferative β-cell phenotype (43, 51, 58). Fold stimulation of insulin secretion thus peaked at the lower doses (1 or 3.3 μM) of harmine, and the highest dose led to the lowest fold stimulation, to a level even below that of DMSO-treated samples for 2 out of 3 donors. These observations indicate that utilizing lower harmine doses in a therapeutic setting may be more advantageous and less likely to induce high levels of circulating insulin under unstimulated conditions, which could contribute to insulin resistance and β-cell exhaustion. Finally, based on the discrepancy between low β-cell and high non-β-cell proliferation rates and increased β-cell fraction upon harmine treatment, it may be that harmine also acts through yet unstudied mechanisms such as transdifferentiation (37, 61). Further studies involving insulin, glucagon, and somatostatin staining, in combination with FACS-sorting methods to generate MTs composed of restricted cell types, would be required to determine whether such transdifferentiation events indeed occur upon harmine treatment.

Our results have led to interesting conclusions regarding the influence of harmine dose on β-cell function and proliferation. Although NKX6.1 staining intensity significantly increased in a dose-dependent manner and showed similar trends after 4 and 15 days of treatment for Donor 3, proliferation and fold-stimulation were reduced in the MTs that received the long-term treatment. Although upon short-term treatment, the highest concentration of harmine tested led to the highest proliferative rates and DAPI counts, harmine’s effect on total cell count, islet volume, β-cell proliferation and fold-stimulation of insulin secretion was maximized at lower doses for long-term treatments, thus supporting the use of lower doses to maximize therapeutic effects.

In summary, our platform enables the parallel evaluation of total cell count, β-cell fraction and mass, β- and non-β-cell proliferation, and β-cell function for the validation, identification, and evaluation of novel β-cell proliferative agents using human islet MTs. Additional multiplexing to include hormone stainings, α-cell quantification, and analysis of α-cell function could be envisioned to obtain even a broader scope of information. The high reproducibility of human islet MTs enables robust assessment of dosing schemes of compounds individually or synergistically and can lead to valuable insights in determining optimal dosing while considering proliferation, function, viability, and toxicity. Furthermore, dissociated islets can be cryopreserved and thawed to generate new islet MTs, permitting the banking and subsequent reuse of the same donor for subsequent experiments. Due to the numerous discrepancies between rodent and human β-cell proliferative capacity as well as the challenges associated with working with native human islets, such a platform should help facilitate the development of novel therapies for both T1D and T2D. Further studies investigating human β-cell proliferation under stress models relevant for T1D and T2D and over longer treatment periods will be needed to fully understand the potential of harmine and other proliferative compounds to regenerate β-cells in diabetic patients.

## Data Availability Statement

The original contributions presented in the study are included in the article/**Supplementary Material**. Further inquiries can be directed to the corresponding author.

## Author Contributions

BY and TK conceptualized the project idea. BY, JM-C, and AT designed the study. AT wrote the manuscript with support from BY, MK, and JM-C. MK, BY, and OG established imaging analysis pipelines, and MK and BY performed image analysis. AT, CR, FF, and SJ performed experiments and analyzed data. SS, AT, and FF established staining protocols. MK, AT, and BY prepared figures. BY and TK acquired funding. All authors have read and approved of the final manuscript.

## Funding

This work was financially supported by InSphero AG and Boehringer Ingelheim. This project has also received funding from the European Eurostars Program under grant agreement E!12988 (“PAN-Kit”).

## Conflict of Interest

AT, MK, JM-C, OG, CR, SS, FF, and SJ are or were employees of InSphero AG, a company commercializing islet MTs and related services. BY is a member of the management team of InSphero AG. TK is an employee of Boehringer Ingelheim, a company active in diabetes treatment and management markets.

## Publisher’s Note

All claims expressed in this article are solely those of the authors and do not necessarily represent those of their affiliated organizations, or those of the publisher, the editors and the reviewers. Any product that may be evaluated in this article, or claim that may be made by its manufacturer, is not guaranteed or endorsed by the publisher.

## Supplementary Material

The Supplementary Material for this article can be found online at: https://figshare.com/s/cd15752aa61a9e6f48e7(DOI 10.6084/m9.figshare.18166688)

Supplementary Figure 1 Assessment of the effects of human islet MT time in culture on proliferation.

Supplementary Figure 2 Assessment of Caspase 3/7 activity in human islet MTs upon harmine treatment.

Supplementary Video 1 3D videos of harmine-treated MTs.

Supplementary Video 2 3D videos of harmine-treated MTs with analysis labels.

Supplementary Video 3 3D render of imaging channels.
